# Metal-tolerant *morganella morganii* isolates can potentially mediate nickel stress tolerance in Arabidopsis by upregulating antioxidative enzyme activities

**DOI:** 10.1080/15592324.2024.2318513

**Published:** 2024-03-25

**Authors:** Tahir Naqqash, Aeman Aziz, Muhammad Baber, Muhammad Shahid, Muhammad Sajid, Radicetti Emanuele, Abdel-Rhman Z. Gaafar, Mohamed S. Hodhod, Ghulam Haider

**Affiliations:** a Institute of Molecular Biology and Biotechnology, Bahauddin Zakariya University, Multan, Pakistan; b Department of Bioinformatics and Biotechnology, Government College University, Faisalabad, Pakistan; c Department of Biotechnology, University of Okara, Okara, Pakistan; d Department of Chemical, Pharmaceutical and Agricultural Sciences, University of Ferrara, Ferrara, Italy; e Department of Botany and Microbiology, College of Science, King Saud University, Riyadh, Saudi Arabia; f Faculty of Biotechnology, October University for Modern Sciences & Arts, 6th October City, Egypt; g Department of Plant Biotechnology, Atta-ur-Rahman School of Applied Biosciences, National University of Sciences and Technology, Islamabad, Pakistan

**Keywords:** Heavy metal, Arabidopsis thaliana, Plant-growth-promoting rhizobacteria, Quantum yield, MDA, SOD

## Abstract

Plant growth-promoting rhizobacteria (PGPRs) have been utilized to immobilize heavy metals, limiting their translocation in metal contaminated settings. However, studies on the mechanisms and interactions that elucidate how PGPRs mediate Nickel (Ni) tolerance in plants are rare. Thus, in this study we investigated how two pre-characterized heavy metal tolerant isolates of *Morganella morganii* (ABT9 and ABT3) improve Ni stress tolerance in Arabidopsis while enhancing its growth and yield. Arabidopsis seedlings were grown for five weeks in control/Ni contaminated (control, 1.5 mM and 2.5 mM) potted soil, in the presence or absence of PGPRs. Plant growth characteristics, quantum yield, and antioxidative enzymatic activities were analyzed to assess the influence of PGPRs on plant physiology. Oxidative stress tolerance was quantified by measuring MDA accumulation in Arabidopsis plants. As expected, Ni stress substantially reduced plant growth (shoot and root fresh weight by 53.25% and 58.77%, dry weight by 49.80% and 57.41% and length by 47.16% and 64.63% over control), chlorophyll content and quantum yield (by 40.21% and 54.37% over control). It also increased MDA content by 84.28% at higher (2.5 mM) Ni concentrations. In contrast, inoculation with *M. morganii* led to significant improvements in leaf chlorophyll, quantum yield, and Arabidopsis biomass production. The mitigation of adverse effects of Ni stress on biomass observed in *M. morganii*-inoculated plants was attributed to the enhancement of antioxidative enzyme activities compared to Ni-treated plants. This upregulation of the antioxidative defense mechanism mitigated Ni-induced oxidative stress, leading to improved performance of the photosynthetic machinery, which, in turn, enhanced chlorophyll content and quantum yield. Understanding the underlying mechanisms of these tolerance-inducing processes will help to complete the picture of PGPRs-mediated defense signaling. Thus, it suggests that *M. morganii* PGPRs candidate can potentially be utilized for plant growth promotion by reducing oxidative stress via upregulating antioxidant defense systems in Ni-contaminated soils and reducing Ni metal uptake.

## Introduction

The occurrence of heavy metals in natural soil resources has become a matter of global concern. Heavy metals can enter the soil through various sources such as mining activities, atmospheric deposition, petrochemical operations, coal combustion, spillage events, sewage sludge, irrigation with wastewater, paint usage, fertilizer utilization, pesticide application, animal manure disposal, and waste management practices, resulting in high metal concentration.^1,2^ Once introduced into the soil, heavy metals persist for prolonged periods, as they are not amenable to biological degradation and can only undergo changes in oxidation state.^3^ Among the various heavy metals, nickel (Ni) is one of the most toxic and widespread pollutants in the soil. This has led to significant reductions in crop yields and negative impacts on plant growth by inhibiting photosynthesis and electron transport processes in plants.^4–6^

The presence of Ni contamination in land poses a serious environmental, economic, and health concern, not only in Pakistan but also in other developing nations.^4^ Utilizing such lands for agricultural purposes requires the implementation of efficient and safe decontamination processes. Despite the availability of various clean-up methods, the extent of contamination and its impact on the environment remain pervasive. To address this challenge, it is imperative to implement site-specific decontamination methods that can achieve optimal results with minimal cost and adverse effects. A feasible approach to mitigate the problem of heavy metal contamination is the utilization of plant growth promoting rhizobacteria (PGPRs) to enhance metal uptake.^7^

In recent years, researchers have explored the use of PGPRs to mitigate the adverse effects of heavy metal stress on plants. These bacteria can colonize the rhizosphere, a region around plant roots, providing various benefits to the plant, including increased tolerance to heavy metals.^8–10^ The remediation of heavy metal contamination by metal-tolerant PGPRs can be achieved through various mechanisms including bio-precipitation, bio-sorption, bio-accumulation, and bio-stabilization.^11,12^

These microorganisms can mediate heavy metal uptake in plants due to the presence of binding ligands, such as polysaccharides and glycoproteins such as mannans, phospho-mannans, glucans, and chitin in their cell walls.^13^ The heavy metals bound to these ligands can then be detoxified through processes like valence transformation, extracellular precipitation of chemicals, and volatilization. Furthermore, plant-beneficial rhizobacteria can counteract the negative effects of toxic metals on different plants through the production of exopolysaccharides, antioxidative enzymes and other growth-improving mechanisms, such as phytohormone production and nutrient uptake.^7,14^

The impact of PGPRs on the phytoremediation of heavy metal contaminated soil has been well documented in the literature. Various genera of rhizobacteria including *Azotobacter*, *Achromobacter*, *Azospirillum*, *Arthrobacter*, *Bacillus*, *Enterobacter*, *Serratia*, and *Pseudomonas* have been found to promote plant growth in metal-polluted soil and to improve the phytoremediation of heavy metal-contaminated land.^15–18^

Despite abundant evidence supporting the positive impact of PGPRs on plant growth, limited attention has been paid to the role of bacteria in the phytoremediation of Ni and its effect on plant growth. Therefore, the present study aims to investigate the potential of metal tolerant *Morganella morganii* PGPRs in inducing Ni tolerance and assess their impact on the growth of Arabidopsis plant. This paper provides valuable insights for researchers interested in exploring the use of PGPRs for mitigating the negative effects of heavy metal pollution in agriculture.

## Materials & methods

The phytoremediation potential of two heavy metal tolerant *Morganella morganii* isolates ABT9 (ON316874) and ABT3 (ON316873) was evaluated in a pot experiment at the Institute of Molecular Biology and Biotechnology, Bahauddin Zakariya University, Multan. The study investigated the effect of these isolates on Arabidopsis growth, antioxidant activity, and photosynthetic efficiency in Ni-contaminated soil. The strains were previously isolated and characterized from a salt-affected region in Pakistan.

## Nickel tolerance of PGPRs isolates

The Ni resistance of ABT9 and ABT3 was screened using the spot inoculation technique.^19^ The pure rhizobacterial isolates were inoculated onto LB media containing various Ni concentrations (1 mM to 5 mM) and incubated at 28 ± 2°C for 24 hours. The minimum inhibitory concentration of Ni was determined as the highest concentration that allowed for growth of both ABT9 and ABT3 cultures.

## Preparation of inoculum for pot experiment

The preparation of the inoculum for both ABT9 and ABT3 strains involved the following steps. The strains were cultivated in 100 mL of LB media within conical flasks and then incubated in a shaking incubator at 28 ± 2°C with agitation at 100 rpm for a period of two days. Subsequently, the cells were collected via centrifugation for five minutes, and the resulting pellet was again suspended in sterile water. The optical density of each strain was determined using a spectrophotometer, with measurements taken at 535 nm, and was maintained at a level of 10^8^ CFU/mL.^20^ The resulting suspended solution was then utilized for the Arabidopsis seeds inoculation.

## Plant inoculation experiment

A pot experiment was conducted using a completely randomized design, in accordance with established protocols. Each of the nine experimental treatments were represented by four independent replicates to ensure robustness of the data obtained. The treatments included water control, *M. morganii* ABT9, *M. morganii* ABT3 inoculation, Ni contamination (0 mM, 1.5 mM & 2.5 mM), and the co-treatment of each Ni contamination with each *M. morganii* metal-tolerant rhizobacterial inoculation. The seeds of *Arabidopsis thaliana* (Columbia-0) were collected from NIBGE (National Institute for Biotechnology and Genetic Engineering) located in Faisalabad, Pakistan. Approximately 100 seeds of *Arabidopsis thaliana* were placed in a 50% (v/v) solution of commercial bleach and sterile distilled water for 5–10 minutes. After thorough rinsing with sterile distilled water and removal of excess water by decanting, the seeds were immersed in the inoculum of each respective strain for 30 minutes.

The sterilized and inoculated seeds were evenly spread on the surface of the sterilized sand and were then placed in a growth chamber maintained at 20–22°C under a 16-hour photoperiod, 8-hour dark period with 75% humidity. After three weeks of germination, a secondary inoculation was administered by incorporating it into the Hoagland nutrient solution.^21^ Five-week-old plants were subjected to Ni treatments, beginning with a concentration of 0.5 mM, which gradually increased to the desired level of 2.5 mM. After the final treatment was administered for a period of two days, the plants were harvested for the determination of agronomic parameters, quantum yield, and chlorophyll content (SPAD). For biochemical assays, the plants were frozen using liquid nitrogen and stored at a temperature of −80°C.

## Agronomic parameters

The fresh biomass of the shoot and root was determined using an accurate weighing balance. The dry biomass was obtained by subjecting the shoot and root samples to a controlled drying process at 65°C for 10 consecutive days. The length of the root and shoot was measured using a precise ruler or scale.

## Chlorophyll content

The chlorophyll (Chl) content was determined using a SPAD-502 chlorophyll meter. In order to obtain a reliable measurement, three separate readings were recorded from four replicates and their mean reading was considered to be the standard one.^22^

## Quantum yield

The quantum yield (F_v_/F_m_) of the Arabidopsis plant was determined using the Flour-Pen FP100 (PSI, CZ) instrument. A standardized procedure was employed, which involved exposing a 5-centimeter area of the plant leaves to a light pulse (3000 µmol m^−2^ s^−1^). The readings were obtained following established protocols, as reported in previous studies.^23^

## MDA

To determine the concentration of malondialdehyde (MDA) in leaves, plant tissue weighing 200 mg was pulverized using a 0.1% trichloroacetic acid (TCA) solution and then subjected to centrifugation at 10,000 rpm for 5 minutes. Subsequently, 1 mL of the extracted enzyme was mixed with 3 mL of 5% TCA solution containing 1% thiobarbituric acid, and the mixture was heated in a water bath at 95°C for 30 minutes. The resulting solution was centrifuged again at 5,000 rpm for 5 minutes, and the supernatant was collected and subjected to spectrophotometric analysis at 532 nm and 600 nm. The concentration of MDA was estimated using 155 μM^−1^ cm^−1^ extinction coefficient.^24^

## Antioxidant enzymes estimation

The leaf tissue samples were collected and homogenized in potassium phosphate buffer (100 mM; pH 7.0) to obtain a homogenate following centrifugation for 15 minutes at 10,000 *g* and 4°C to obtain the supernatant, which was used as the enzyme source.^25^

## Measurement of catalase activity

The catalase (CAT) activity was estimated by determining the rate of hydrogen peroxide (H_2_O_2_) decomposition. The 200 µL of enzyme extract was mixed thoroughly with 1.5 mL of 50 mM Na_3_ PO_4_ (7.8 pH) and H_2_O_2_ (300 µL) to a final concentration of 0.3 mM and the decrease in absorbance at 240 nm was measured over time up to 1 min.^26^

## Measurement of peroxidase activity

The peroxidase (POD) activity was determined by measuring the rate of guaiacol oxidation. The supernatant (50 µL) was mixed with 1 mL of 50 mM Na_3_ PO_4_ (7.8 pH), 20 mM guaiacol (0.2 mL) and 40 mM H_2_O_2_ (0.25 mL) and the increase in absorbance at 470 nm was measured over time up to 2 min.^26^

## Measurement of superoxide dismutase activity

The superoxide dismutase (SOD) activity was evaluated using the photoreduction assay of Nitro Blue Tetrazolium (NBT).^27^ A reaction mixture was prepared by mixing 33 mM NBT (50 μL), enzyme extract (100 μL), 0.003 mM riboflavin (50 μL), 10 mM L-methionine (100 μL), and 50 mM potassium phosphate buffer (250 μL). The reaction was initiated by exposing the mixture to light from a 15 W lamp for 30 minutes, followed by termination of the reaction by turning off the light. Control experiments were conducted, both in the presence and absence of light, with the omission of the enzyme extract. Absorbance was measured at 560 nm, and the concentration of SOD required to inhibit 50% of the NBT reduction was defined as 1 unit activity of SOD.

## Statistical analysis

A one-way analysis of variance was performed on the collected data to determine the statistical significance of the results. The mean values of each treatment were then compared using the Fisher’s least significant difference test, which was calculated at a probability level of 5% using Statistix 8.1 software. The level of significance was determined as follows: *p* ≤ 0.001 (***); *p* ≤ 0.005 (**); and *p* ≤ 0.5 (*). Origin-Pro 2021 software was used for principal component analysis (PCA).

## Results

The impact of two Ni-tolerant *Morganella morganii* strains (ABT9 and ABT3) on the growth characteristics of Arabidopsis seedlings subjected to Ni stress was evaluated by measuring shoot length, root length, fresh weight, and dry weight of the seedlings.

**Figure 1.**
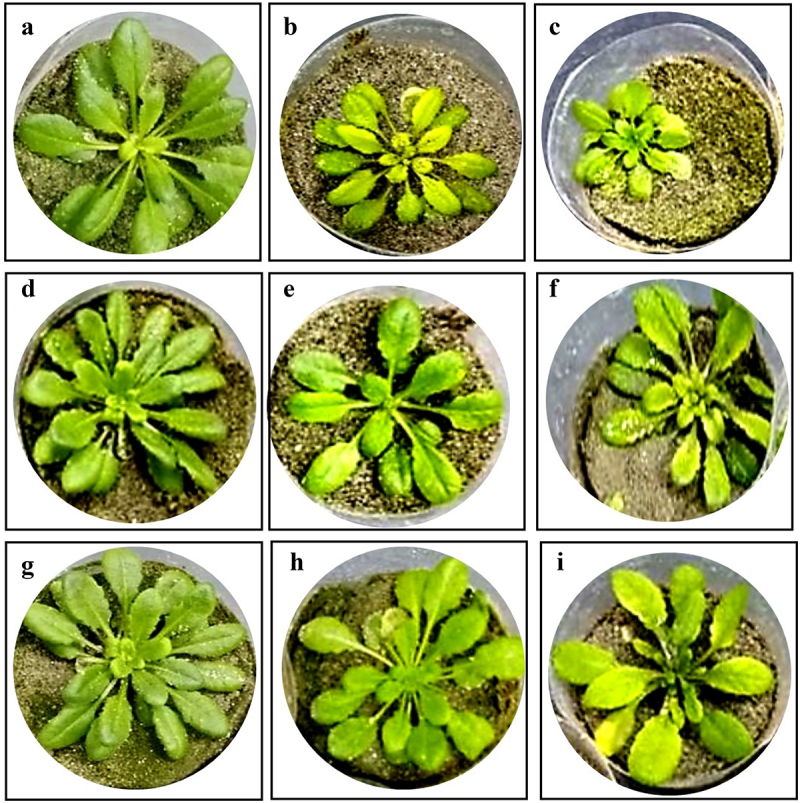
Representative picture of Arabidopsis plant under Ni stress (a) water control (b) 1.5 mM Ni, (c) 2.5 mM Ni, (d) *M. morganii* ABT3 inoculation, (e) *M. morganii* ABT3 + 1.5 mM Ni, (f) *M. morganii* ABT3 + 2.5 mM Ni, (g) *M. morganii* ABT9 inoculation, (h) *M. morganii* ABT9 + 1.5 mM Ni, (i) *M. morganii* ABT9 + 2.5 mM Ni.

**Figure 2.**
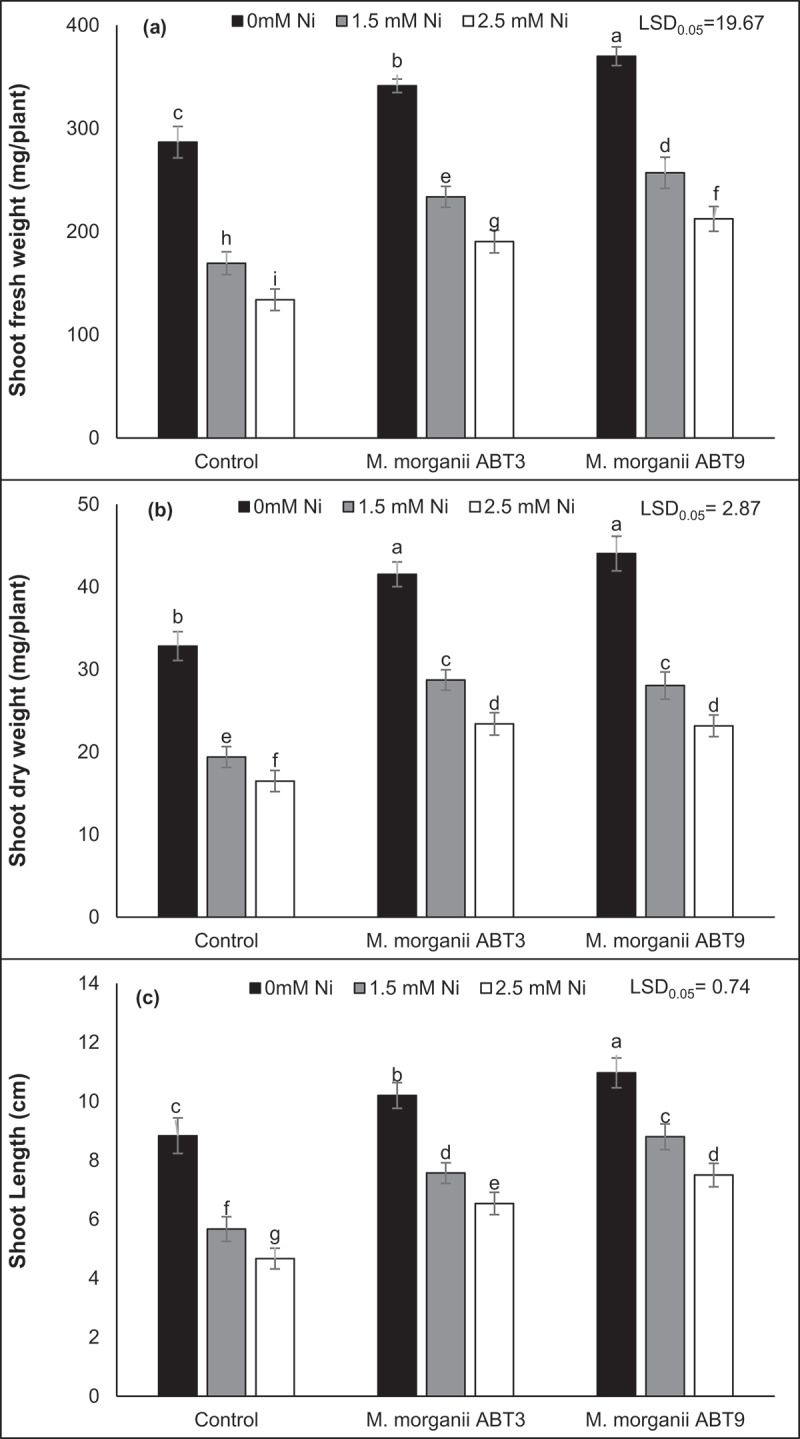
Effect of Ni-tolerant *morganella morganii* isolates inoculation on Arabidopsis growth and dry matter production in nickel-contaminated soil. The three pieces of figure represent, (a) fresh weight; (b) dry weight; and the (c) shoot length of plants. The vertical bars are based on the means of four biological replicates and the error bars represent standard deviation. The bars sharing similar letters are not significantly different at *p* = 0.05%.

Results of the pot experiment indicated that Ni contamination resulted in a statistically significant decrease in the fresh weight, dry weight and length of the shoot compared to the uninoculated control plants of Arabidopsis (Figures 1 and 2). The maximum reduction in shoot fresh weight (53.25%), dry weight (49.80%) and length (47.16%) was observed in the Arabidopsis plants under 2.5 mM Ni stress compared to the uninoculated controls. However, the results showed that the utilization of Ni-tolerant *M. morganii* (ABT9 and ABT3) isolates caused a significant increase in shoot biomass in Arabidopsis plant under both levels of Ni contamination. Notably, the ABT9 strain exhibited the highest improvement in shoot fresh weight and length with an increase of 58.45% and 60.71%, respectively, while the ABT3 isolate showed maximum improvement in shoot dry weight with an increase of 42.03% under 2.5 mM Ni stress conditions, compared to non-inoculated Arabidopsis seedlings grown under the same stress level.

**Figure 3.**
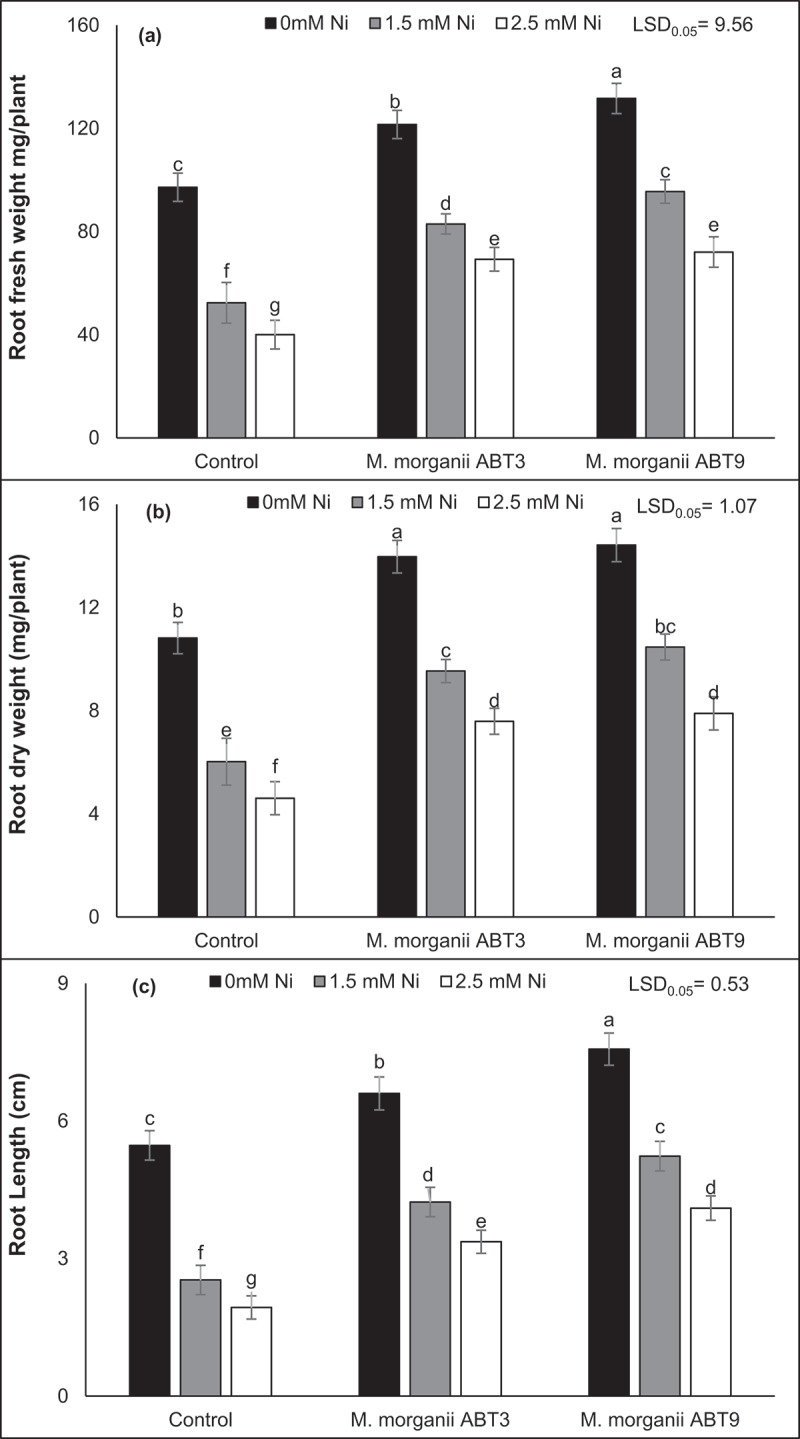
Effect of Ni-tolerant *morganella morganii* isolates inoculation on Arabidopsis root growth and dry matter production in nickel-contaminated soil. The figure pieces represent, (a) fresh weight of roots; (b) dry weight of roots; and overall (c) root length. The vertical bars are based on the means of four biological replicates and the error bars represent standard deviation. The bars sharing similar letters are not significantly different at *p* = 0.05%.

In a similar manner, Ni contamination caused a statistically significant reduction in the root length, fresh weight, and dry weight of Arabidopsis plants compared to the uninoculated control plants (Figure 3). The decline in root length and fresh and dry weight biomass increased as the Ni concentration increased. The maximum reduction in root fresh weight (58.77%), dry weight (57.41%) and length (64.63%) was observed in the Arabidopsis plants under 2.5 mM Ni stress compared to the uninoculated controls.

It has been observed that the introduction of Ni-tolerant strains of *M. morganii* (ABT9 and ABT3) had a positive impact on the root biomass at both Ni levels. The Ni-tolerant ABT9 rhizobacterium was found to significantly enhance the root fresh weight, dry weight, and length by 79.86%, 71.42%, and 112.06%, respectively, compared to control plants of Arabidopsis that were grown under 2.5 mM Ni contamination without any inoculation

**Figure 4.**
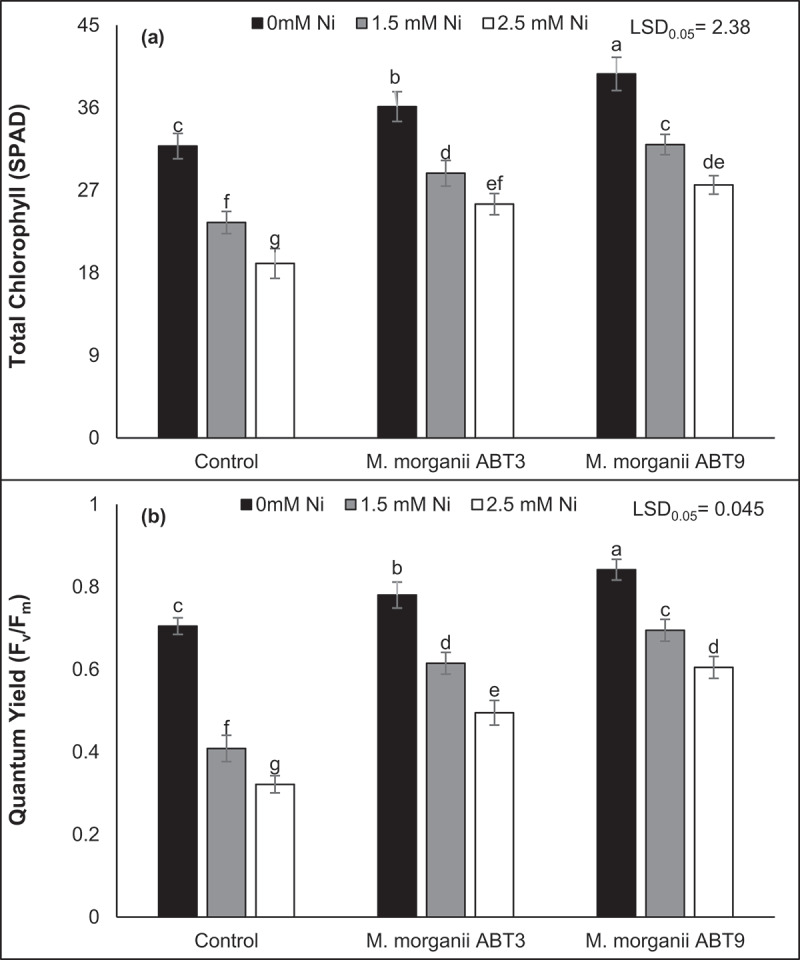
Effect of Ni-tolerant *morganella morganii* isolates inoculation on Arabidopsis photosynthetic efficiency grown in nickel-contaminated soil. The figure parts represent, (a) total chlorophyll content and photosynthetic efficiency as (b) quantum yield. The vertical bars are based on the means of four biological replicates and the error bars represent standard deviation. The bars sharing similar letters are not significantly different at *p* = 0.05%.

Furthermore, the influence of Ni-tolerant isolates of *M. morganii* (ABT9 and ABT3) on the photosynthetic efficiency of Arabidopsis plants was assessed by determining total Chl content and F_v_/F_m_ (quantum yield). These measurements provided insight into the effect of the inoculation on both the overall pigment content and primary photochemical reactions of the Arabidopsis plants. The results of the study showed that the presence of Ni stress resulted in a substantial decrease in both total Chl content and F_v_/F_m_. A decrease of 40.21% and 54.37% was observed in the total Chl content and F_v_/F_m_, respectively in of seedlings exposed to a 2.5 mM Ni concentration, when compared to the control seedlings (Figure 4). On the contrary, inoculation with Ni-tolerant *M. morganii* (ABT9 and ABT3) isolates had a positive effect on these parameters, demonstrating significant enhancement. The results showed that the use of Ni-tolerant ABT9 rhizobacterium resulted in the most substantial improvements total Chl content (45.02%) and F_v_/F_m_ (88.08%) compared to control plants of Arabidopsis grown under 2.5 mM Ni contamination without inoculation. These findings suggest the potential of Ni-tolerant rhizobacteria in mitigating the negative effects of Ni stress on photosynthetic efficiency in Arabidopsis plants.

**Figure 5.**
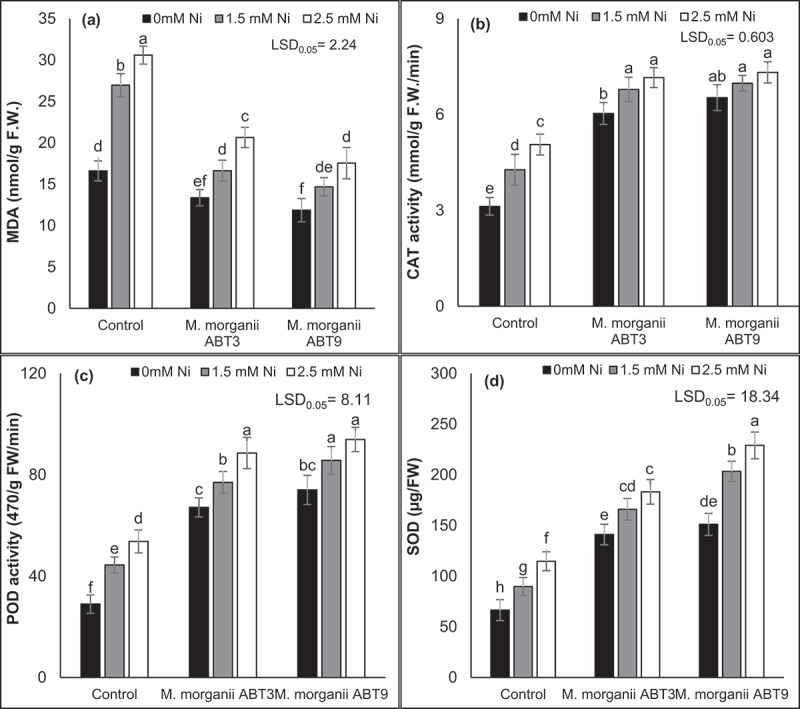
Effect of Ni-tolerant *morganella morganii* isolates inoculation on oxidative stress in Arabidopsis grown in nickel-contaminated soil. The figure pieces represent the activities of different antioxidant enzymes, such as: (a) MDA; (b) CAT; (c) POD; and (d) SOD. The vertical bars are based on the means of four biological replicates and the error bars represent standard deviation. The bars sharing similar letters are not significantly different at *p* = 0.05%.

The results of the study indicated that Ni stress resulted in a noticeable increase in lipid peroxidation and the antioxidative enzymatic activities, including SOD, POD, and CAT in comparison to unstressed control Arabidopsis plants (Figure 5). The highest elevations in MDA, SOD, POD, and CAT were observed at a Ni concentration of 2.5 mM, with increments of 84.28%, 72.66%, 85.40%, and 61.94%, respectively, in comparison to control seedlings without inoculation. The inoculation of Ni-tolerant *M. morganii* (ABT9 and ABT3) isolates was found to mitigate the detrimental effects of Ni stress on lipid peroxidation by causing a considerable increase in the antioxidative enzymatic activities in Ni-stressed Arabidopsis plants. At a Ni concentration of 1.5 mM, both strains (ABT9 and ABT3) resulted in the reduction of lipid peroxidation by 45.53% and 38.31%, respectively, while at 2.5 mM Ni, reductions of 42.64% and 32.52, respectively, were observed. At both Ni contaminations (1.5 mM & 2.5 mM), the maximum improvement in the activities of SOD, POD, and CAT was observed in Arabidopsis plants inoculated with the ABT9 strain, with increments of 126.76% and 99.70% for SOD, 92.94% and 72.92% for POD, and 63.34% and 44.59% for CAT, respectively, in comparison to the respective control seedlings without inoculation.

**Figure 6.**
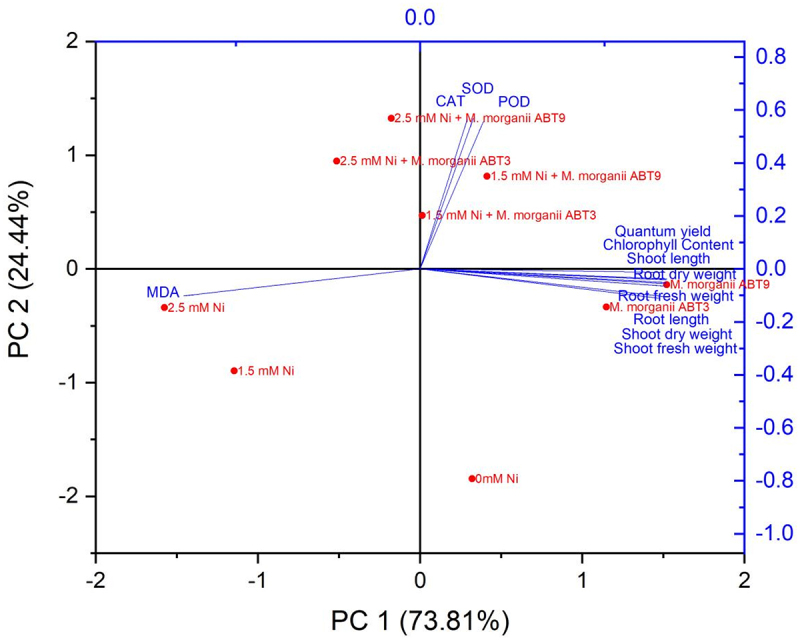
Principal component analysis plot illustrating the effect of Ni-tolerant *Morganella morganii* isolates inoculation on biomass, photosynthetic parameters, and antioxidative enzymatic activities in Arabidopsis plants grown under Ni stress.

The PCA results revealed the phytoremediation potential of treatments using Ni-tolerant *M. morganii* (ABT9 and ABT3) isolates in Arabidopsis plants exposed to Ni contamination. The distribution of the nine treatments along PC1 and PC2 demonstrated the efficacy of the Ni-tolerant isolates (Figure 6; Supplementary Table 1). PC1 accounted for a significant portion (73.81%) to the variance in the data, while PC2 accounted for 24.44% of the variance. PC1 comprised of both inoculated treatments, with or without Ni stress, and a positive association was noted in all growth parameters, quantum yield, total Chl content and antioxidative enzymatic activities of the Arabidopsis seedlings, except for lipid peroxidation. These results demonstrate the presence of a positive relationship between the inoculated treatments and the mentioned parameters, which suggests the effectiveness of Ni-tolerant rhizobacterial isolates in promoting phytoremediation in contaminated environments.

## Discussion

In recent years, the application of metal-tolerant PGPRs has been extensively employed in the field of phytoremediation for metal-contaminated soils. PGPRs play a vital function in promoting the growth of plants and reducing the toxicity of various heavy metals, thereby making them a valuable tool in the bioremediation of contaminated areas.^28,29^ Therefore, this study investigated the potential of Ni-tolerant *M. morganii* isolates to alleviate the negative effects of nickel stress on *Arabidopsis thaliana*.

A plethora of research studies has established the fact that excessive heavy metals in the growth environment of plants can result in detrimental effects, disrupting metabolic processes of the plant and inhibiting development and growth.^30,31^ Furthermore, previous studies have indicated that metal toxicity can result in a reduction of shoot and root lengths in certain plant species. For example, *Zea mays* subjected to Pb and Cu,^32^ *Vigna mungo* subjected to Cd,^33^ *Pennisetum purpureum* subjected to Pb,^34^ and *Oryza sativa* subjected to As^35^ have all reported a decline in shoot and root biomass. This suppression is believed to be associated with a direct inhibition of root and shoot metabolic processes.^36^

Similar reduction in growth and length of shoot and root was observed in the Arabidopsis plants in this study. The reduced plant growth observed in the presence of Ni could potentially be attributed to a decrease in water potential and an imbalance in nutrient availability. However, one of the main results of the study was that the Ni-tolerant *M. morganii* isolates promoted the biomass of Arabidopsis shoot and root under nickel contamination. PGPRs have been found to exert a significant positive impact on plant growth by facilitating the essential nutrients uptake by promoting microbial turnover. This turnover induces different metabolic activities that are involved in the mineralization and solubilization of organic phosphorus, leading to an increase in plant growth. Studies have demonstrated that the inoculation of metal tolerant PGPRs can stimulate roots and shoot biomass in plants under metal toxicity. For example, inoculation with *Enterobacter* has been shown to elevate the growth characteristics of Cd-exposed *O. sativa*.^37^ Inoculation with *Bacillus* sp. also resulted in higher shoot and root biomass in Cd-exposed *O. sativa* plants.^38^ In addition, *Trichoderma* sp. has been found to improve plant growth characteristics under As toxicity, with As-tolerant strains enhancing phosphate solubilization and improving the metabolic and physiological activities of *Cicer arietinum*.^39^ These results suggest that the PGPRs strains have the potential to enhance biomass production under heavy metal stress.

The findings of the current investigation indicate that Ni stress decreased the photosynthetic pigment and quantum yield in Arabidopsis seedlings. Previous research has also demonstrated that exposure to heavy metal disrupts the photosynthetic machinery in rice and wheat plants by altering the chlorophyll pigments and net photosynthetic activity, leading to structural changes in leaves that impair their photosynthetic apparatus.^40,41^ The reduction in photosynthetic pigments has been primarily caused by the loss of cell wall and membrane integrity, particularly of the thylakoid membrane. Additionally, heavy metals can inhibit enzymes activity involved in chlorophyll synthesis that leads to the breakdown of chlorophyll pigments, which is crucial for the photosynthetic process.^42^ The reduction in chlorophyll content could result in damage to Photosystem II (PSII) due to the retrogression of the D1 protein, which ultimately inhibits synthesis of chlorophyll and decrease quantum yield.^43^ Our findings are consistent with previous study in which a decline in chlorophyll and quantum yield was observed in Cd-treated *Brassica juncea* and *Brassica campestris*.^44^

The presence of PGPRs could lead to an increase in photosynthetic pigments, as these microorganisms promote nutrient uptake via solubilization of phosphate and the exudation of crucial substances required for the synthesis of photosynthetic pigments necessary for photo-assimilation and light-harvesting complexes.^45,46^ *Klebsiella pneumoniae* supplementation in *V. mungo* increased chlorophyll levels and F_v_/F_m_ under Cd stress.^33^ Additionally, *Azotobacter chroococcum* supplementation in Pb and Cu exposed plants of *Zea mays* led to an increase in chlorophyll content and overall net photosynthetic efficiency.^32^ The increase could be due to the growth promoting effects of PGPRs, as well as the increased protein levels that stimulate pigment synthesis.

Membrane damage caused by oxidative stress can lead to the accumulation of malondialdehyde, an oxidized form resulting disintegrated membrane lipids due to lipid peroxidation as well as oxidative burst.^47^ The contamination of zinc in maize plants has been shown to increase electrolyte leakage and ROS accumulation, indicative of a loss of membrane integrity due to oxidative stress.^48^ Similar observations were made in earlier studies, which reported that Cd and Ni stress resulted in lipid peroxidation.^49,50^ Furthermore, an increase in MDA content has been documented in response to Cr, Zn and Cu, which is consistent with the results of present study.^51–53^ In this study, MDA level was reduced following supplementation with the Ni-tolerant *M. morganii* isolates, which could be attributed to the Ni oxidative stress mitigation by the microbes. This finding is in line with the previous report, which suggested that *Funneliformis mosseae* reduced MDA levels in *Capsicum annum* under copper stress.^54^ The reduction of MDA levels was also reported in Ni-stressed *Vinca rosea* in the presence of *Bacillus megaterium*.^55^

Additionally, the application of metal-tolerant PGPRs in Cd- and Zn-stressed *Cajanus cajan* reduced MDA contents, which might be attributed to the activation of antioxidative enzyme activities that mitigate ROS and membrane damage.^56,57^ The reduction in MDA content may be due to the *M. morganii*-mediated immobilization and exclusion, which decreases the effective concentration of metals and indirectly alleviates the inhibitory effect of Ni-induced oxidative stress on enzymatic activities. However, the activity of antioxidant enzymes in metal-stressed plants can vary greatly depending on the type of metal, species, exposure duration, and concentration reflecting the altered redox status of stressed cells.^58^

In general, the oxidative damage in plants is controlled by both enzymatic and non-enzymatic components. Superoxide dismutase is an essential component of the antioxidative defense system that rapidly converts superoxide radicals to hydrogen peroxide, protecting cells against the toxic effects of reactive oxygen species.^59^ An increase in SOD activity is usually associated with higher concentrations of superoxide radicals, likely due to de novo synthesis of the enzyme.^59,60^ In this study, *M. morganii*-inoculated plants grown in soil amended with Ni had higher SOD activity, suggesting that inoculation increased the plants’ ability to produce SOD as observed previously.^61,62^

Another critical component of the ROS antioxidative process is CAT, which breaks down hydrogen peroxide into water.^63^ The activity of CAT can either increase or decrease under metal stress, depending on the specific conditions.^64,65^ In our study, CAT activity increased significantly in *M. morganii*-inoculated plants under Ni stress, paralleling the enhanced activity of SOD and indicating the induction of oxidative stress tolerance. Similarly, the activity of POD was also enhanced in plants grown in Ni-contaminated soil inoculated with bacteria and in plants grown in Ni-contaminated soil alone, which is consistent with previous research work.^55,66^ It appears that all antioxidative enzymes work together under *M. morganii* inoculation, contributing to better growth under such conditions, suggesting that PGPRs inoculation reduced oxidative stress in Arabidopsis plants. However, more experiments are needed to implement more specific mechanistic approaches at the molecular level. Exploring variations in proline content, ROS content, and defense hormones between inoculated and non-inoculated treatments would add an interesting dimension to the study. This understanding holds significant relevance, particularly in the context of heavy metal-tolerant isolates like *M. morganii*, given their potential application in heavy metal detoxification.

## Conclusions

This study highlights the role of Ni tolerant PGPRs strains of *M. morganii* in promoting plant growth and alleviating Ni toxicity in Arabidopsis seedlings. The inoculation of *M. morganii* strains led to an increase in growth which can be attributed to the *M. morganii*-mediated nutrient availability, such as phosphorus and nitrogen. This nutrient availability could alleviate the detrimental effects of Ni stress on nutrient uptake. Additionally, the activity of antioxidant enzymes SOD, POD, and CAT were enhanced, further supporting the role of PGPRs in mitigating Ni toxicity by reducing oxidative stress (MDA content), resulting in an increase in chlorophyll content and quantum yield. Our findings suggest that the use of Ni-tolerant PGPRs (*M. morganii*) holds great potential for improving the growth and photosynthetic activity of plants grown in Ni contaminated soils. This approach could be a sustainable and eco-friendly strategy for remediating heavy metal contaminated soils. Future research should focus on gaining a deeper understanding of the molecular mechanisms behind heavy metal detoxification and to examine the effects of bacterial inoculation on soil biological activities in different environmental conditions.

## Supplementary Material

Supplementary Information.docx

## Data Availability

The data reported in the present study are deposited in the NCBI repository under the accession numbers: ON316874 and ON316873.
