# Supplementary material for: Metal-tolerant morganella morganii isolates can potentially mediate nickel stress tolerance in Arabidopsis by upregulating antioxidative enzyme activities
Source: Plant Signal Behav. 2024 Mar 25;19(1):2318513. doi: 10.1080/15592324.2024.2318513 (PMC10965111; doi:10.1080/15592324.2024.2318513)
Supplement: Supplementary Information.docx [file KPSB_A_2318513_SM5701.docx]

**Supplementary Information**

**Metal-tolerant *Morganella morganii* isolates can potentially mediate Nickel stress tolerance in Arabidopsis by upregulating antioxidative enzyme activities**

Tahir Naqqash ^1*^, Aeman Aziz^1^, Muhammad Baber^1^, Muhammad Shahid^2^, Muhammad Sajid^3^, Radicetti Emanuele^4^, Abdel-Rhman Z. Gaafar^5^, Mohamed S. Hodhod^6^, Ghulam Haider^7*^

^1^Institute of Molecular Biology and Biotechnology, Bahauddin Zakariya University, Multan 60800, Pakistan.

^2^Department of Bioinformatics and Biotechnology, Government College University, Faisalabad 38000, Pakistan.

^3^Department of Biotechnology, University of Okara, Okara, Pakistan.

^4^Department of Chemical, Pharmaceutical and Agricultural Sciences, University of Ferrara, Via Luigi Borsari n. 46, 44121 Ferrara, Italy.

^5^Department of Botany and Microbiology, College of Science, King Saud University, Riyadh, 11451, Saudi Arabia

^6^Faculty of Biotechnology, October University for Modern Sciences & Arts, 6^th^ October City, 12566, Egypt

^7^Department of Plant Biotechnology, Atta-ur-Rahman School of Applied Biosciences, National University of Sciences and Technology, 44000 Islamabad, Pakistan

**Table S1. PCA analysis results showing the percentage variance of Arabidopsis plants for different treatments.**

| **PC 1 (73.81%)** | **PC 2 (24.44%)** | **Treatments** |
| --- | --- | --- |
| 0.32027 | -1.8457 | 0mM Ni |
| -1.14692 | -0.89744 | 1.5 mM Ni |
| -1.57631 | -0.34131 | 2.5 mM Ni |
| 1.14795 | -0.33523 | *M. morganii* ABT3 |
| 1.52068 | -0.13892 | *M. morganii* ABT9 |
| 0.01297 | 0.47029 | 1.5 mM Ni + *M. morganii* ABT3 |
| 0.41339 | 0.81542 | 1.5 mM Ni + *M. morganii* ABT9 |
| -0.51406 | 0.94841 | 2.5 mM Ni + *M. morganii* ABT3 |
| -0.17798 | 1.32449 | 2.5 mM Ni + *M. morganii* ABT9 |
| **PC 1 (73.81%)** | **PC 2 (24.44%)** | **Parameters** |
| 0.32976 | -0.10393 | Shoot fresh weight |
| 0.32392 | -0.11127 | Shoot dry weight |
| 0.33398 | -0.05096 | Root fresh weight |
| 0.33228 | -0.06459 | Root dry weight |
| 0.33445 | -0.03759 | Shoot length |
| 0.33328 | -0.05561 | Root length |
| 0.33386 | -0.04076 | Chlorophyll Content |
| 0.33242 | -0.01257 | Quantum yield |
| -0.32071 | -0.10231 | MDA |
| 0.08557 | 0.55597 | CAT |
| 0.07215 | 0.56877 | POD |
| 0.06404 | 0.56627 | SOD |
